# 2-(1*H*-indol-3-ylcarbon­yl)acetonitrile

**DOI:** 10.1107/S1600536809001342

**Published:** 2009-02-04

**Authors:** P. Ramesh, A. Subbiahpandi, P. Thirumurugan, Paramasivan T. Perumal, M. N. Ponnuswamy

**Affiliations:** a Department of Physics, Presidency College (Autonomous), Chennai 600 005, India; b Organic Chemistry Division, Central Leather Research Institute, Adyar, Chennai 600 020, India; c Centre of Advanced Study in Crystallography and Biophysics, University of Madras, Guindy Campus, Chennai 600 025, India

## Abstract

The title compound, C_11_H_8_N_2_O, crystallizes with two crystallographically independent mol­ecules in the asymmetric unit which are approximately perpendicular to each other [79.97 (6)°]. The indole ring system is planar [r.m.s. deviation = 0.010 (1) Å]. The crystal structure is stabilized by inter­molecular C—H⋯N and N—H⋯O inter­actions.

## Related literature

For the use of indole derivatives as bioactive drugs, see: Stevenson *et al.* (2000 ▶). For their biological properties, see: Harris & Uhle (1960 ▶); Ho *et al.* (1986 ▶). For their high aldose reductase inhibitory activity, see: Rajeswaran *et al.* (1999 ▶). For a related structure, see: Ramesh *et al.* (2008 ▶). For hydrogen-bond motifs, see: Bernstein *et al.* (1995 ▶).
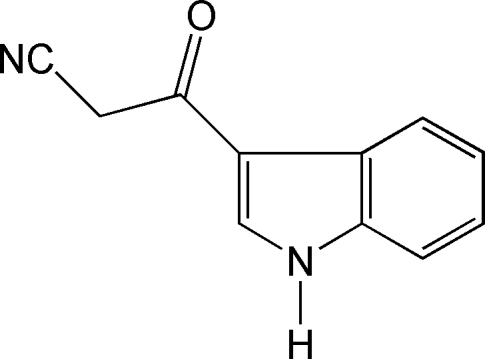


## Experimental

### 

#### Crystal data

C_11_H_8_N_2_O
*M* *_r_* = 184.19
Triclinic, 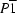

*a* = 7.3439 (2) Å
*b* = 7.3534 (2) Å
*c* = 18.2475 (5) Å
α = 83.402 (2)°
β = 78.890 (2)°
γ = 73.501 (1)°
*V* = 925.23 (4) Å^3^
*Z* = 4
Mo *K*α radiation
μ = 0.09 mm^−1^
*T* = 293 (2) K
0.30 × 0.30 × 0.20 mm

#### Data collection

Bruker Kappa APEXII area-detector diffractometer
Absorption correction: multi-scan (*SADABS*; Sheldrick, 2001 ▶) *T*_min_ = 0.974, *T*_max_ = 0.983
18838 measured reflections
3253 independent reflections
2717 reflections with *I* > 2σ(*I*)
*R*_int_ = 0.025

#### Refinement

*R*[*F* ^2^ > 2σ(*F* ^2^)] = 0.037
*wR*(*F* ^2^) = 0.100
*S* = 1.06
3253 reflections
253 parameters
H-atom parameters constrained
Δρ_max_ = 0.12 e Å^−3^
Δρ_min_ = −0.16 e Å^−3^

### 

Data collection: *APEX2* (Bruker, 2004 ▶); cell refinement: *SAINT* (Bruker, 2004 ▶); data reduction: *SAINT*; program(s) used to solve structure: *SHELXS97* (Sheldrick, 2008 ▶); program(s) used to refine structure: *SHELXL97* (Sheldrick, 2008 ▶); molecular graphics: *ORTEP-3* (Farrugia, 1997 ▶); software used to prepare material for publication: *SHELXL97* and *PLATON* (Spek, 2003 ▶).

## Supplementary Material

Crystal structure: contains datablocks global, I. DOI: 10.1107/S1600536809001342/bt2839sup1.cif

Structure factors: contains datablocks I. DOI: 10.1107/S1600536809001342/bt2839Isup2.hkl

Additional supplementary materials:  crystallographic information; 3D view; checkCIF report

## Figures and Tables

**Table 1:** Hydrogen-bond geometry (Å, °)

*D*—H⋯*A*	*D*—H	H⋯*A*	*D*⋯*A*	*D*—H⋯*A*
N1—H1⋯O1^i^	0.86	1.98	2.8146 (18)	163
C2—H2⋯N13^i^	0.93	2.59	3.236 (2)	127
N1′—H1′⋯O1′^ii^	0.86	2.00	2.8166 (15)	158

Symmetry codes: (i) 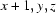; (ii) 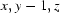.

**Fig. 1.**
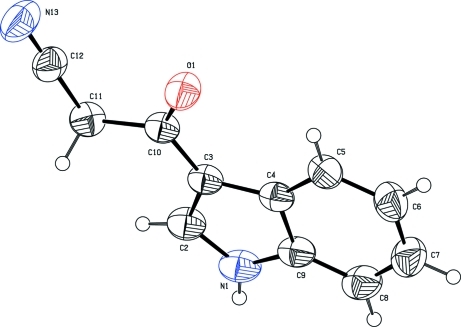
Perspective view of the molecule showing the thermal ellipsoids are drawn at 50% probability level. The H atoms are shown as small circles of arbitrary radii.

**Fig. 2.**
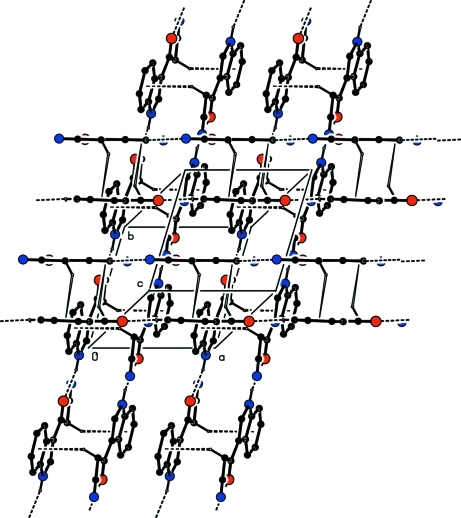
The crystal packing of the molecules viewed down c axis. H atoms not involved in hydrogen bonding have been omitted for clarity.

## supplementary crystallographic information

### Comment

Indole derivatives are used as bioactive drugs (Stevenson *et al.*, 2000) and they exibit anti-allergic, central nervous system depressant and muscle relaxant properties (Harris & Uhle 1960; Ho *et al.*, 1986). Indoles have been proved to display high aldose reductase inhibitory activity (Rajeswaran *et al.*, 1999). Against this background and to ascertain the molecular conformation, the structure determination of the title compound has been carried out.

There are two crystallographically independent molecules in the asymmetric unit and they are approximately perpendicular to each other [79.97 (6)°]. In both molecules the indole ring systems are planar and the sum of the angles at N1 (359.95°) and N1' (359.94°) are in accordance with *sp^2^* hybridization. The bond angles of the cyano group   [179.8 (2) and 179.6 (2)°] in both molecules show  their linear   character. The C≡N bond distances [1.133 (2)  and 1.131 (2) Å] are comparable with the literature values (Ramesh *et al.*, 2008).

The crystal packing is covered by C—H···N, C—H···O and C—H···π types intermolecular interactions in addition to van der Waals forces. N1 atom in one of the molecules   donates one proton to O1 (-1 + *x*, *y*, *z*) which connects the molecules into a one dimensional C6 chain (Bernstein *et al.*, 1995) running along the *a* axis, whereas in the other molecule at N1'  forms similar network with O1' (*x*, 1 + *y*, *z*) along the *b* axis. The combination of N1—H1···O1 and C2—H2···N13 hydrogen bonds form a *R*_2_^2^ (9) dimer chain running along the *a* axis.

### Experimental

Indole (5.85 g, 50 mmol) was added to a solution prepared by dissolution of cyanoacetic acid (5.0 g, 50 mmol) in Ac_2_O (50 ml) at 50 °C. The solution was heated at 85 °C for 5 min. During that period 3-cyanoacetylindole started to crystallize. After 5 more min, the mixture was allowed to cool and the solid was collected, washed with MeOH, and dried.

### Refinement

H atoms were positioned geometrically (N—H = 0.86 Å, and C—H = 0.93–0.97 Å) and allowed to ride on their parent atoms, with *U*_iso_(H) = 1.5*U*_eq_(C) for methyl H, 1.2*U*_eq_(C) for other H atoms.

### Crystal data

**Table:**

C_11_H_8_N_2_O	*Z* = 4
*M**_r_* = 184.19	*F*(000) = 384
Triclinic, *P*1	*D*_x_ = 1.322 Mg m^−^^3^
Hall symbol: -P 1	Mo *K*α radiation, λ = 0.71073 Å
*a* = 7.3439 (2) Å	Cell parameters from 3554 reflections
*b* = 7.3534 (2) Å	θ = 1.1–25.0°
*c* = 18.2475 (5) Å	µ = 0.09 mm^−^^1^
α = 83.402 (2)°	*T* = 293 K
β = 78.890 (2)°	Block, colourless
γ = 73.501 (1)°	0.30 × 0.30 × 0.20 mm
*V* = 925.23 (4) Å^3^	

### Data collection

**Table:**

Bruker Kappa APEXII area-detector diffractometer	3253 independent reflections
Radiation source: fine-focus sealed tube	2717 reflections with *I* > 2σ(*I*)
graphite	*R*_int_ = 0.025
ω and φ scans	θ_max_ = 25.0°, θ_min_ = 1.1°
Absorption correction: multi-scan (*SADABS*; Sheldrick, 2001)	*h* = −7→8
*T*_min_ = 0.974, *T*_max_ = 0.983	*k* = −8→8
18838 measured reflections	*l* = −21→21

### Refinement

**Table:**

Refinement on *F*^2^	Primary atom site location: structure-invariant direct methods
Least-squares matrix: full	Secondary atom site location: difference Fourier map
*R*[*F*^2^ > 2σ(*F*^2^)] = 0.037	Hydrogen site location: inferred from neighbouring sites
*wR*(*F*^2^) = 0.100	H-atom parameters constrained
*S* = 1.06	*w* = 1/[σ^2^(*F*_o_^2^) + (0.0404*P*)^2^ + 0.2437*P*] where *P* = (*F*_o_^2^ + 2*F*_c_^2^)/3
3253 reflections	(Δ/σ)_max_ = 0.008
253 parameters	Δρ_max_ = 0.12 e Å^−^^3^
0 restraints	Δρ_min_ = −0.16 e Å^−^^3^

### Special details

**Table:**

Geometry. All e.s.d.'s (except the e.s.d. in the dihedral angle between two l.s. planes) are estimated using the full covariance matrix. The cell e.s.d.'s are taken into account individually in the estimation of e.s.d.'s in distances, angles and torsion angles; correlations between e.s.d.'s in cell parameters are only used when they are defined by crystal symmetry. An approximate (isotropic) treatment of cell e.s.d.'s is used for estimating e.s.d.'s involving l.s. planes.
Refinement. Refinement of *F*^2^ against ALL reflections. The weighted *R*-factor *wR* and goodness of fit *S* are based on *F*^2^, conventional *R*-factors *R* are based on *F*, with *F* set to zero for negative *F*^2^. The threshold expression of *F*^2^ > σ(*F*^2^) is used only for calculating *R*-factors(gt) *etc*. and is not relevant to the choice of reflections for refinement. *R*-factors based on *F*^2^ are statistically about twice as large as those based on *F*, and *R*- factors based on ALL data will be even larger.

### Fractional atomic coordinates and isotropic or equivalent isotropic displacement parameters (Å^2^)

**Table:**

	*x*	*y*	*z*	*U*_iso_*/*U*_eq_	
O1	0.13478 (16)	0.2464 (2)	1.01403 (7)	0.0659 (4)	
O1'	0.76317 (17)	0.33368 (14)	0.47980 (6)	0.0543 (3)	
N1	0.7573 (2)	0.2397 (2)	1.00692 (9)	0.0619 (4)	
H1	0.8678	0.2314	1.0183	0.074*	
N1'	0.77718 (19)	−0.28849 (17)	0.48288 (7)	0.0479 (3)	
H1'	0.7932	−0.4024	0.4709	0.057*	
C2	0.6023 (2)	0.2202 (2)	1.05570 (11)	0.0567 (4)	
H2	0.5977	0.1956	1.1071	0.068*	
C2'	0.8188 (2)	−0.1468 (2)	0.43593 (9)	0.0441 (4)	
H2'	0.8689	−0.1567	0.3853	0.053*	
C3	0.4501 (2)	0.2418 (2)	1.01850 (9)	0.0475 (4)	
C3'	0.7768 (2)	0.01550 (19)	0.47349 (8)	0.0385 (3)	
C4	0.5195 (2)	0.2786 (2)	0.94094 (9)	0.0478 (4)	
C4'	0.7012 (2)	−0.03211 (19)	0.54959 (8)	0.0389 (3)	
C5'	0.6267 (2)	0.0675 (2)	0.61400 (9)	0.0479 (4)	
H5'	0.6214	0.1956	0.6133	0.058*	
C5	0.4391 (2)	0.3141 (2)	0.87583 (10)	0.0565 (4)	
H5	0.3107	0.3175	0.8777	0.068*	
C6'	0.5613 (3)	−0.0269 (2)	0.67853 (9)	0.0569 (4)	
H6'	0.5098	0.0389	0.7216	0.068*	
C6	0.5521 (3)	0.3437 (3)	0.80899 (11)	0.0678 (5)	
H6	0.4991	0.3687	0.7653	0.081*	
C7'	0.5702 (3)	−0.2187 (2)	0.68090 (10)	0.0583 (4)	
H7'	0.5269	−0.2792	0.7257	0.070*	
C7	0.7456 (3)	0.3370 (3)	0.80531 (13)	0.0742 (6)	
H7	0.8195	0.3559	0.7591	0.089*	
C8'	0.6417 (2)	−0.3200 (2)	0.61849 (10)	0.0527 (4)	
H8'	0.6475	−0.4483	0.6199	0.063*	
C8	0.8287 (3)	0.3032 (3)	0.86844 (13)	0.0684 (5)	
H8	0.9574	0.2993	0.8660	0.082*	
C9	0.7140 (2)	0.2751 (2)	0.93550 (11)	0.0540 (4)	
C9'	0.7049 (2)	−0.2241 (2)	0.55332 (8)	0.0419 (3)	
C10	0.2612 (2)	0.2273 (2)	1.05110 (9)	0.0482 (4)	
C10'	0.8024 (2)	0.19645 (19)	0.44251 (8)	0.0396 (3)	
C11'	0.8851 (2)	0.2118 (2)	0.35992 (8)	0.0455 (4)	
H11A	1.0210	0.1451	0.3524	0.055*	
H11B	0.8214	0.1505	0.3322	0.055*	
C11	0.2210 (2)	0.1842 (3)	1.13506 (9)	0.0562 (4)	
H11C	0.3072	0.0628	1.1480	0.067*	
H11D	0.2474	0.2809	1.1602	0.067*	
C12	0.0245 (3)	0.1779 (3)	1.16141 (9)	0.0605 (5)	
C12'	0.8620 (2)	0.4067 (2)	0.33086 (9)	0.0513 (4)	
N13	−0.1297 (3)	0.1725 (3)	1.18193 (9)	0.0878 (6)	
N13'	0.8429 (3)	0.5597 (2)	0.30820 (9)	0.0806 (5)	

### Atomic displacement parameters (Å^2^)

**Table:**

	*U*^11^	*U*^22^	*U*^33^	*U*^12^	*U*^13^	*U*^23^
O1	0.0436 (6)	0.1027 (10)	0.0585 (7)	−0.0291 (6)	−0.0166 (6)	0.0039 (7)
O1'	0.0829 (8)	0.0340 (6)	0.0491 (6)	−0.0248 (5)	−0.0035 (6)	−0.0054 (5)
N1	0.0409 (8)	0.0647 (9)	0.0868 (11)	−0.0155 (7)	−0.0217 (8)	−0.0100 (8)
N1'	0.0611 (8)	0.0298 (6)	0.0566 (8)	−0.0163 (6)	−0.0112 (6)	−0.0059 (6)
C2	0.0469 (9)	0.0597 (10)	0.0689 (11)	−0.0150 (8)	−0.0187 (8)	−0.0087 (8)
C2'	0.0521 (9)	0.0379 (8)	0.0453 (8)	−0.0158 (6)	−0.0082 (7)	−0.0056 (6)
C3	0.0412 (8)	0.0451 (9)	0.0602 (10)	−0.0126 (7)	−0.0144 (7)	−0.0072 (7)
C3'	0.0432 (8)	0.0323 (7)	0.0435 (8)	−0.0136 (6)	−0.0109 (6)	−0.0027 (6)
C4	0.0420 (8)	0.0367 (8)	0.0674 (11)	−0.0124 (6)	−0.0128 (7)	−0.0038 (7)
C4'	0.0409 (8)	0.0333 (7)	0.0461 (8)	−0.0130 (6)	−0.0123 (6)	−0.0011 (6)
C5'	0.0566 (9)	0.0388 (8)	0.0501 (9)	−0.0144 (7)	−0.0095 (7)	−0.0043 (7)
C5	0.0509 (9)	0.0527 (10)	0.0689 (11)	−0.0184 (8)	−0.0160 (8)	0.0048 (8)
C6'	0.0671 (11)	0.0573 (10)	0.0458 (9)	−0.0172 (8)	−0.0067 (8)	−0.0043 (8)
C6	0.0761 (13)	0.0590 (11)	0.0706 (13)	−0.0260 (9)	−0.0147 (10)	0.0103 (9)
C7'	0.0656 (11)	0.0587 (11)	0.0514 (10)	−0.0237 (8)	−0.0092 (8)	0.0103 (8)
C7	0.0741 (13)	0.0641 (12)	0.0810 (14)	−0.0287 (10)	0.0052 (11)	0.0041 (10)
C8'	0.0611 (10)	0.0385 (8)	0.0623 (11)	−0.0213 (7)	−0.0151 (8)	0.0093 (7)
C8	0.0486 (10)	0.0584 (11)	0.0983 (16)	−0.0215 (8)	−0.0032 (10)	−0.0021 (10)
C9	0.0424 (9)	0.0429 (9)	0.0794 (12)	−0.0144 (7)	−0.0114 (8)	−0.0055 (8)
C9'	0.0443 (8)	0.0348 (7)	0.0498 (9)	−0.0136 (6)	−0.0119 (7)	−0.0016 (6)
C10	0.0438 (8)	0.0472 (9)	0.0576 (10)	−0.0131 (7)	−0.0148 (7)	−0.0065 (7)
C10'	0.0432 (8)	0.0347 (7)	0.0446 (8)	−0.0140 (6)	−0.0106 (6)	−0.0026 (6)
C11'	0.0512 (9)	0.0421 (8)	0.0451 (9)	−0.0155 (7)	−0.0081 (7)	−0.0026 (7)
C11	0.0554 (10)	0.0621 (10)	0.0551 (10)	−0.0163 (8)	−0.0154 (8)	−0.0085 (8)
C12	0.0591 (12)	0.0765 (12)	0.0430 (9)	−0.0117 (9)	−0.0078 (8)	−0.0100 (8)
C12'	0.0641 (10)	0.0487 (10)	0.0432 (9)	−0.0227 (8)	−0.0044 (8)	0.0001 (7)
N13	0.0640 (11)	0.1429 (18)	0.0513 (10)	−0.0231 (11)	−0.0009 (8)	−0.0111 (10)
N13'	0.1215 (15)	0.0564 (10)	0.0620 (10)	−0.0331 (10)	−0.0029 (10)	0.0065 (8)

### Geometric parameters (Å, °)

**Table:**

O1—C10	1.2190 (18)	C5—H5	0.9300
O1'—C10'	1.2173 (17)	C6'—C7'	1.389 (2)
N1—C2	1.335 (2)	C6'—H6'	0.9300
N1—C9	1.379 (2)	C6—C7	1.396 (3)
N1—H1	0.8600	C6—H6	0.9300
N1'—C2'	1.3372 (19)	C7'—C8'	1.367 (2)
N1'—C9'	1.3755 (19)	C7'—H7'	0.9300
N1'—H1'	0.8600	C7—C8	1.371 (3)
C2—C3	1.379 (2)	C7—H7	0.9300
C2—H2	0.9300	C8'—C9'	1.381 (2)
C2'—C3'	1.373 (2)	C8'—H8'	0.9300
C2'—H2'	0.9300	C8—C9	1.376 (3)
C3—C10	1.429 (2)	C8—H8	0.9300
C3—C4	1.431 (2)	C10—C11	1.517 (2)
C3'—C10'	1.4331 (19)	C10'—C11'	1.516 (2)
C3'—C4'	1.436 (2)	C11'—C12'	1.445 (2)
C4—C5	1.393 (2)	C11'—H11A	0.9700
C4—C9	1.406 (2)	C11'—H11B	0.9700
C4'—C5'	1.393 (2)	C11—C12	1.443 (3)
C4'—C9'	1.3987 (19)	C11—H11C	0.9700
C5'—C6'	1.372 (2)	C11—H11D	0.9700
C5'—H5'	0.9300	C12—N13	1.133 (2)
C5—C6	1.369 (3)	C12'—N13'	1.132 (2)
			
C2—N1—C9	109.94 (14)	C8'—C7'—H7'	119.4
C2—N1—H1	125.0	C6'—C7'—H7'	119.4
C9—N1—H1	125.0	C8—C7—C6	121.35 (19)
C2'—N1'—C9'	109.56 (12)	C8—C7—H7	119.3
C2'—N1'—H1'	125.2	C6—C7—H7	119.3
C9'—N1'—H1'	125.2	C7'—C8'—C9'	117.34 (15)
N1—C2—C3	109.81 (16)	C7'—C8'—H8'	121.3
N1—C2—H2	125.1	C9'—C8'—H8'	121.3
C3—C2—H2	125.1	C7—C8—C9	117.29 (18)
N1'—C2'—C3'	110.07 (14)	C7—C8—H8	121.4
N1'—C2'—H2'	125.0	C9—C8—H8	121.4
C3'—C2'—H2'	125.0	C8—C9—N1	130.03 (16)
C2—C3—C10	126.55 (16)	C8—C9—C4	122.72 (17)
C2—C3—C4	106.56 (14)	N1—C9—C4	107.25 (15)
C10—C3—C4	126.89 (14)	N1'—C9'—C8'	129.55 (14)
C2'—C3'—C10'	126.66 (14)	N1'—C9'—C4'	107.66 (13)
C2'—C3'—C4'	106.34 (12)	C8'—C9'—C4'	122.78 (14)
C10'—C3'—C4'	126.99 (13)	O1—C10—C3	122.51 (15)
C5—C4—C9	118.60 (16)	O1—C10—C11	119.87 (15)
C5—C4—C3	134.95 (15)	C3—C10—C11	117.61 (13)
C9—C4—C3	106.45 (14)	O1'—C10'—C3'	122.74 (13)
C5'—C4'—C9'	118.54 (13)	O1'—C10'—C11'	120.06 (13)
C5'—C4'—C3'	135.07 (13)	C3'—C10'—C11'	117.20 (12)
C9'—C4'—C3'	106.36 (12)	C12'—C11'—C10'	112.33 (13)
C6'—C5'—C4'	118.70 (14)	C12'—C11'—H11A	109.1
C6'—C5'—H5'	120.6	C10'—C11'—H11A	109.1
C4'—C5'—H5'	120.6	C12'—C11'—H11B	109.1
C6—C5—C4	118.94 (17)	C10'—C11'—H11B	109.1
C6—C5—H5	120.5	H11A—C11'—H11B	107.9
C4—C5—H5	120.5	C12—C11—C10	112.34 (14)
C5'—C6'—C7'	121.46 (16)	C12—C11—H11C	109.1
C5'—C6'—H6'	119.3	C10—C11—H11C	109.1
C7'—C6'—H6'	119.3	C12—C11—H11D	109.1
C5—C6—C7	121.09 (19)	C10—C11—H11D	109.1
C5—C6—H6	119.5	H11C—C11—H11D	107.9
C7—C6—H6	119.5	N13—C12—C11	179.8 (2)
C8'—C7'—C6'	121.15 (16)	N13'—C12'—C11'	179.6 (2)
			
C9—N1—C2—C3	0.0 (2)	C2—N1—C9—C4	0.35 (18)
C9'—N1'—C2'—C3'	0.34 (17)	C5—C4—C9—C8	−0.7 (2)
N1—C2—C3—C10	178.76 (15)	C3—C4—C9—C8	179.28 (15)
N1—C2—C3—C4	−0.41 (19)	C5—C4—C9—N1	179.39 (14)
N1'—C2'—C3'—C10'	179.69 (14)	C3—C4—C9—N1	−0.59 (17)
N1'—C2'—C3'—C4'	−0.69 (17)	C2'—N1'—C9'—C8'	179.31 (16)
C2—C3—C4—C5	−179.36 (17)	C2'—N1'—C9'—C4'	0.18 (17)
C10—C3—C4—C5	1.5 (3)	C7'—C8'—C9'—N1'	−177.93 (15)
C2—C3—C4—C9	0.61 (17)	C7'—C8'—C9'—C4'	1.1 (2)
C10—C3—C4—C9	−178.56 (15)	C5'—C4'—C9'—N1'	177.78 (13)
C2'—C3'—C4'—C5'	−177.20 (16)	C3'—C4'—C9'—N1'	−0.59 (16)
C10'—C3'—C4'—C5'	2.4 (3)	C5'—C4'—C9'—C8'	−1.4 (2)
C2'—C3'—C4'—C9'	0.78 (16)	C3'—C4'—C9'—C8'	−179.80 (14)
C10'—C3'—C4'—C9'	−179.60 (14)	C2—C3—C10—O1	−179.17 (16)
C9'—C4'—C5'—C6'	0.4 (2)	C4—C3—C10—O1	−0.2 (3)
C3'—C4'—C5'—C6'	178.23 (16)	C2—C3—C10—C11	0.1 (2)
C9—C4—C5—C6	0.2 (2)	C4—C3—C10—C11	179.09 (15)
C3—C4—C5—C6	−179.88 (17)	C2'—C3'—C10'—O1'	−179.31 (15)
C4'—C5'—C6'—C7'	0.8 (3)	C4'—C3'—C10'—O1'	1.1 (2)
C4—C5—C6—C7	0.6 (3)	C2'—C3'—C10'—C11'	−0.3 (2)
C5'—C6'—C7'—C8'	−1.2 (3)	C4'—C3'—C10'—C11'	−179.83 (13)
C5—C6—C7—C8	−0.9 (3)	O1'—C10'—C11'—C12'	−14.8 (2)
C6'—C7'—C8'—C9'	0.2 (3)	C3'—C10'—C11'—C12'	166.12 (13)
C6—C7—C8—C9	0.3 (3)	O1—C10—C11—C12	−2.2 (2)
C7—C8—C9—N1	−179.67 (17)	C3—C10—C11—C12	178.56 (15)
C7—C8—C9—C4	0.5 (3)	C10—C11—C12—N13	51 (88)
C2—N1—C9—C8	−179.50 (18)	C10'—C11'—C12'—N13'	−58 (25)

### Hydrogen-bond geometry (Å, °)

**Table:**

*D*—H···*A*	*D*—H	H···*A*	*D*···*A*	*D*—H···*A*
N1—H1···O1^i^	0.86	1.98	2.8146 (18)	163
C2—H2···N13^i^	0.93	2.59	3.236 (2)	127
N1'—H1'···O1'^ii^	0.86	2.00	2.8166 (15)	158

Symmetry codes: (i) *x*+1, *y*, *z*; (ii) *x*, *y*−1, *z*.
